# Liver-specific 3D sectioning molds for correlating *in vivo* CT and MRI with tumor histopathology in woodchucks (*Marmota monax*)

**DOI:** 10.1371/journal.pone.0230794

**Published:** 2020-03-26

**Authors:** Andrew S. Mikhail, Michal Mauda-Havakuk, Ari Partanen, John W. Karanian, William F. Pritchard, Bradford J. Wood

**Affiliations:** Center for Interventional Oncology, Radiology and Imaging Sciences, Clinical Center, National Institutes of Health, Bethesda, Maryland, United States of America; University of Pittsburgh, UNITED STATES

## Abstract

**Purpose:**

To evaluate the spatial registration and correlation of liver and tumor histopathology sections with corresponding *in vivo* CT and MRI using 3D, liver-specific cutting molds in a woodchuck (*Marmota monax*) hepatic tumor model.

**Methods:**

Five woodchucks chronically infected with woodchuck hepatitis virus following inoculation at birth and with confirmed hepatic tumors were imaged by contrast enhanced CT or MRI. Virtual 3D liver or tumor models were generated by segmentation of in vivo CT or MR imaging. A specimen-specific cavity was created inside a block containing cutting slots aligned with an imaging plane using computer-aided design software, and the final cutting molds were fabricated using a 3D printer. Livers were resected two days after initial imaging, fixed with formalin or left unfixed, inserted into the 3D molds, and cut into parallel pieces by passing a sharp blade through the parallel slots in the mold. Histopathology sections were acquired and their spatial overlap with in vivo image slices was quantified using the Dice similarity coefficient (DSC).

**Results:**

Imaging of the woodchucks revealed heterogeneous hepatic tumors of varying size, number, and location. Specimen-specific 3D molds provided accurate co-localization of histopathology of whole livers, liver lobes, and pedunculated tumors with in vivo CT and MR imaging, with or without tissue fixation. Visual inspection of histopathology sections and corresponding in vivo image slices revealed spatial registration of analogous pathologic features. The mean DSC for all specimens was 0.83+/-0.05.

**Conclusion:**

Use of specimen-specific 3D molds for en bloc liver dissection provided strong spatial overlap and feature correspondence between in vivo image slices and histopathology sections.

## Introduction

Hepatocellular carcinoma (HCC) is the most common primary hepatic malignancy and has been increasing in incidence and mortality in the United States for several decades [1]. Imaging-based classification algorithms for hepatic lesions have been developed to standardize HCC diagnoses by associating characteristic imaging features with tumor histopathology [2]. There is a growing need to expand these classifications to include imaging features indicative of molecular, immunologic, and genomic tumor characteristics to better inform prognoses and selection of targeted therapies non-invasively. Such tissue-to-image correlations may also grow in importance with the emergence of artificial intelligence (AI) models for image-based detection and classification tasks [3]. However, few techniques exist for accurate co-localization of imaging with histopathology specimens in the liver, especially in a setting of significant intratumoral heterogeneity.

Advances in computational, feature-based detection algorithms have enabled rapid extraction of large amounts of quantitative data from medical images. Correlating this radiomic data with tumor pathology and treatment outcomes could aid in prospective diagnosis and treatment decisions [4–6]. The success of such analyses largely depends on the ability to accurately co-localize gross- and histo-pathologic specimens with in vivo imaging. However, since the orientation of explanted tissues relative to imaging planes is lost upon resection, dissection of en bloc specimens (“grossing” or “bread loafing”) invariably results in discordance between imaging slices and histopathology.

Several generic reusable sectioning devices have been used to divide specimens into tissue blocks with consistent thickness and approximate anatomic orientation [7–9]. The use of rapid prototyping technology to generate patient-specific molds was originally described by Shah et al for co-localization of prostatectomy specimen histopathology with in vivo magnetic resonance imaging (MRI) [10]. Similar approaches have been used to create 3D-printed cutting molds or sectioning boxes for co-localization of tissue or histopathology with imaging based on in vivo MRI of the prostate [11–18] and kidney [19] and ex vivo MRI of the brain [20]. Recently, 3D molds were also used to facilitate correlation of spatial drug levels following hepatic chemoembolization with radiopaque embolic bead density measured on computed tomography (CT) in frozen liver specimens using a preclinical model [21].

Co-localization of gross- and histo-pathology specimens with in vivo imaging is particularly challenging for highly deformable, variably shaped organs such as the liver, since changes in specimen morphology may complicate alignment with imaging and uniform sectioning. Trout et al. sectioned pediatric hepatectomy specimens using 3D-printed single-sided cutting boxes, each with a recessed contour that matched the surface anatomy of the liver based on preoperative imaging [22]. The aim of our study was to evaluate a method for spatial co-localization of in vivo MRI or CT with gross- and histo-pathology of the liver using fully-encapsulating specimen-specific 3D cutting molds based on the in vivo imaging. This approach is demonstrated in an autochthonous HCC model in woodchucks chronically infected with woodchuck hepatitis virus, which resembles human hepatitis B virus, a leading cause for HCC in humans worldwide [23, 24].

## Materials and methods

### Animal model

This study was carried out in strict accordance with the recommendations in the Guide for the Care and Use of Laboratory Animals of the National Institutes of Health. The protocol was approved by the Animal Care and Use Committee of the NIH Clinical Center (Protocol Number: DRD 16–03). Five woodchucks (Marmota monax) (Northeastern Wildlife, Harrison, Idaho, USA) were studied: three male and two female (average weight = 3.05 kg, range 2.45–3.4; average age = 22.9 months, range 19.5–24.7). Each was chronically infected with woodchuck hepatitis virus following inoculation within the first week of life and confirmed as tumor-positive based on liver enzyme levels and/or diagnostic ultrasound. Woodchucks were individually housed with 12-hour light:dark cycling, ad libitum access to food and water, and environmental enrichment. Cage-side observations were performed twice-daily and woodchucks received a physical assessment immediately prior to imaging procedures. A physical assessment was additionally performed on the first day of the two day housing period following survival imaging procedures.

### Imaging

Woodchucks were sedated using 5% isoflurane via an induction chamber followed by a mixture of pre-anesthetics (28.6 mg/kg ketamine HCl and 5 mg/kg xylazine IM) and then maintained under general anesthesia with 1–5% isoflurane and 100% oxygen (2 L/min) delivered via face mask for the duration of the procedure. Under anesthesia, the woodchucks were shaved and a 21G or 23G angiocatheter was inserted into a forepaw vein for intravenous access during imaging. Multidetector CT imaging was performed (Philips Brilliance MX8000 IDT 16-section Detector CT; Philips, Cleveland, OH, USA). Following non-contrast multidetector CT of the chest and liver, multiphase imaging of the liver was performed with power injection (Medrad Stellant CT Injection System, Bayer Healthcare, Berlin, Germany) of 3.0 mL of iopamidol (Isovue-370, Bracco Diagnostics, Milan, Italy) followed by 3.0 mL 0.9% saline all at 0.2 mL/sec. CT bolus tracking measured in the distal thoracic aorta was used to initiate multiphasic imaging acquired in the early arterial (4 sec delay), late arterial (23 sec delay), portal venous (43 sec delay), late (63 sec delay), and delayed (2 min delay) phases. Scans were obtained at 120 kVp and a tube current of 225 mA with a 180 cm field of view and image reconstruction of 0.8 mm sections at 0.4 mm intervals. A clinical MRI scanner (Achieva 3.0T, Philips, Best, The Netherlands) with a standard 32-channel cardiac RF receive coil was used for MR image acquisition. The coil was immobilized and consisted of 16 elements below the animal and another 16 elements above the animal. MRI pulse sequences included 3D T1 High Resolution Isotropic Volume Excitation (THRIVE), 3D T2-weighted turbo spin echo, dynamic contrast-enhanced, and diffusion-weighted imaging with 1.5 mm slice thickness, 145x145x120 mm field of view, and 0.65x0.65 mm in plane resolution.

After imaging, three woodchucks were housed for two days prior to euthanasia and tissue collection. Two woodchucks were euthanized immediately after imaging. All woodchucks were euthanized by administration of a combination of pentobarbital sodium 390 mg/mL and phenytoin sodium 50 mg/mL (Euthasol 1 mL/10 lb; Virbac Animal Health, Fort Worth, TX, USA) or by exsanguination while under general anesthesia.

### Design and fabrication of liver- and tumor- specific 3D molds

**Fig 1:**
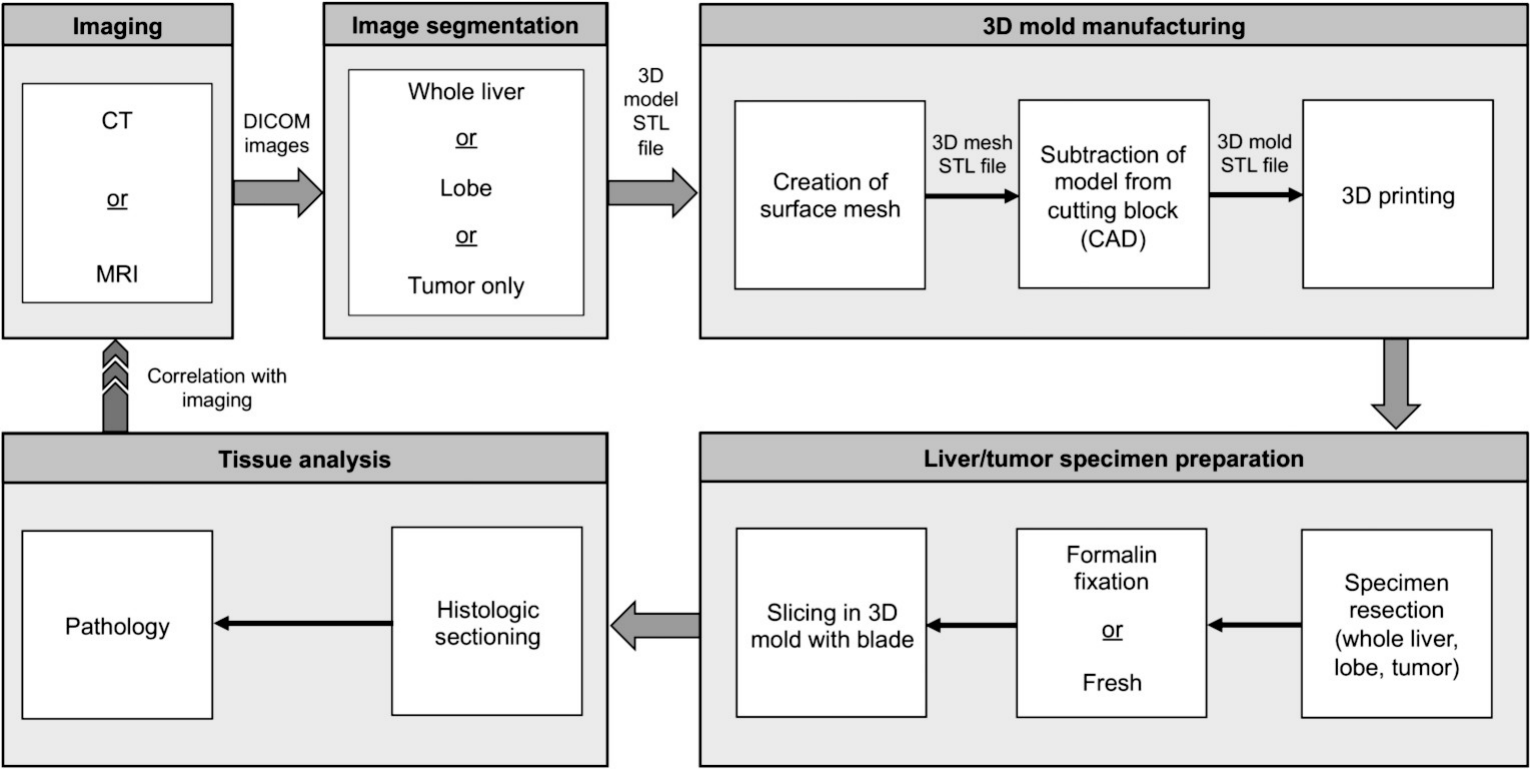
Schematic of procedural workflow. Correlating in vivo imaging with histopathology using liver- or tumor- specific 3D cutting molds.

**Fig 2:**
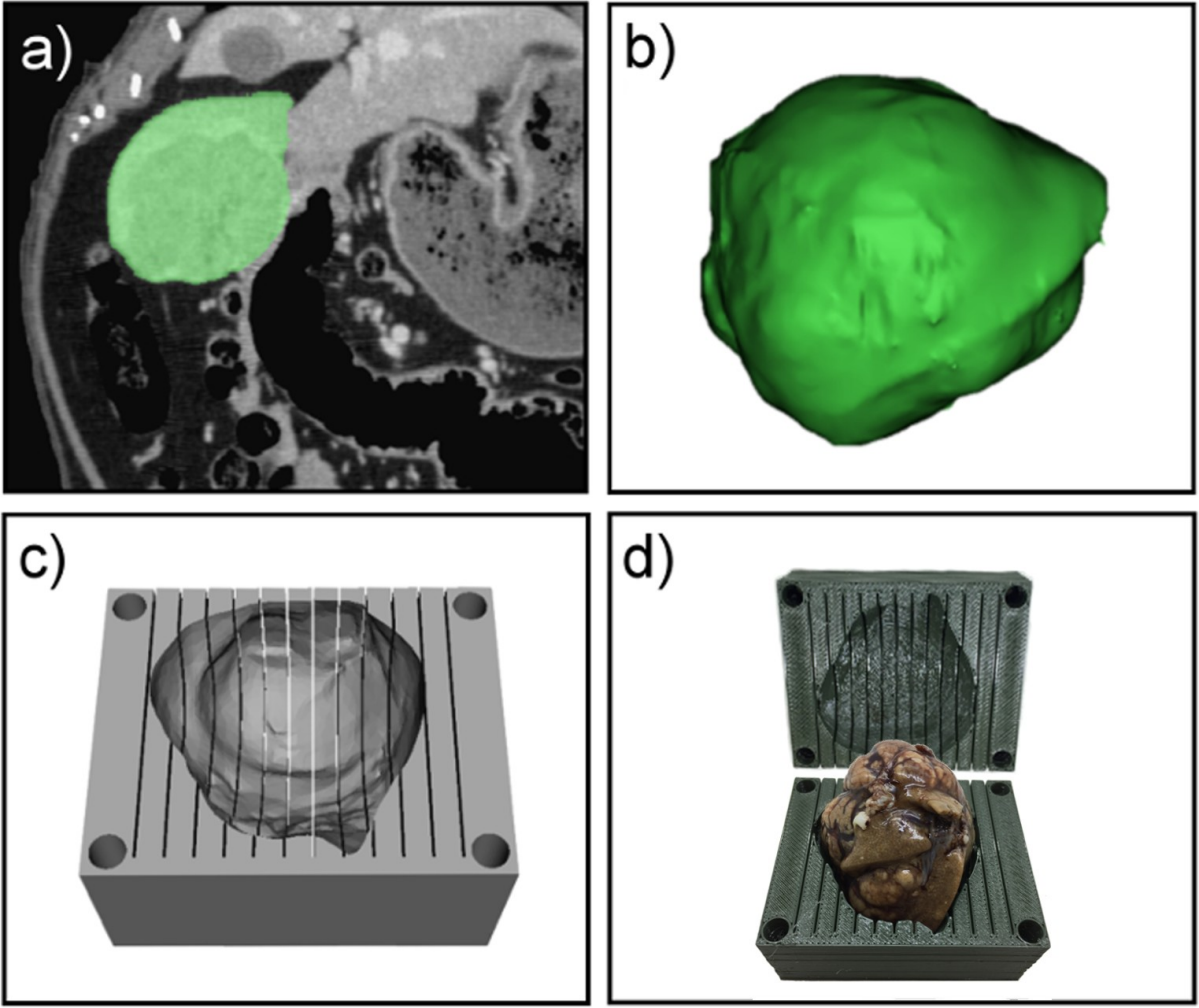
Fabrication of a tumor-specific 3D cutting mold for sectioning liver tumor specimens. (A) Tumor segmentation (green) overlaid on coronal CT, portal venous phase; (B) 3D model of segmented pedunculated tumor; (C) computer-aided design of mold (one side) with tumor-specific cavity and cutting slots in the axial plane; (D) formalin-fixed tumor inserted into the cavity within the tumor-specific 3D cutting mold resulting in alignment of the specimen with the axial CT imaging plane.

Specimen-specific 3D molds were designed and fabricated for correlation of in vivo imaging with histopathology following a modular workflow (Fig 1). Using the in vivo contrast-enhanced CT or MRI, whole livers, liver lobes, or tumors were manually segmented and the resulting mask was converted into a model and a surface wrap was applied using Mimics Research (version 20.0; Materialise, Leuven, Belgium) (Fig 2A and 2B). The models were exported as stereolithography (STL) files and converted to a surface mesh (comprised of vertices and surfaces) using MeshLab (Visual Computing Lab—ISTI—CNR, http://meshlab.sourceforge.net). The number of vertices in the mesh was reduced and the final model was exported as an STL file.

A solid rectangular block was created with 0.5 mm wide parallel cutting slots spaced 5 mm apart with screw holes in each corner using computer-aided design software (SolidWorks, Dassault Système SolidWorks, Concord, MA). The dimensions of the block were adjusted based on the size of specimen to be sectioned. The specimen model STL file was imported as a solid body and a custom cavity unique to each specimen was created by subtraction of the model from the rectangular block. The specimen model was oriented within the block such that the cutting slots corresponded to a standard orthogonal imaging plane, i.e., axial, coronal or sagittal, maintaining the orientation relationship between the imaging and cutting planes. The resulting mold was divided into two separate parts, in proportions that facilitated insertion of the specimen and efficient 3D printing (Fig 2C). Each mold was exported as two STL files, one for each part, and fabricated on a 3D printer (uPrint Plus, Stratasys, Eden Prairie, MN, USA) using acrylonitrile butadiene styrene plastic and soluble support material. After printing, support material was removed from the mold by bathing in a recirculating hot water bath and the specimen was inserted into the mold cavity (Fig 2D).

### Sectioning of liver tumor specimens

For tumors embedded in the liver parenchyma and confined to a single lobe, the entire lobe (N = 1) was resected in order to facilitate correspondence with image segmentation. Pedunculated tumors (N = 2) that were not contained within surrounding liver parenchyma were resected at the stalk without the liver lobe. For comprehensive image to pathology correlation of liver lesions in multiple lobes, the entire liver was segmented and sectioned (N = 2). Specimens were immersed in 10% formalin and stored at 4°C until sectioning (N = 2) or were placed in the mold immediately after euthanasia and sectioned without fixation (N = 3). Specimens were sectioned by passing a single 102 mm sharp carbon steel tissue slicing blade (Thomas Scientific, Swedesboro, NJ) through the slots in the mold. During sectioning, both sides of the mold were held together in place by aligning the screw holes or applying pressure using vise grips. After sectioning, the tissue slabs were placed in standard (1.1x1.6x0.25in) or large (3x2x0.75in) cassettes (Leica Biosystems Inc., Richmond, IL, USA) in a known orientation and immersed in fresh 10% formalin.

### Histopathology

Fixed specimens were processed through a series of increasing concentrations of ethanol, cleared in xylenes and then infiltrated with paraffin wax. Tissues were then embedded in paraffin blocks for sectioning. Sequential parallel sections were cut with 5-μm thickness, mounted on glass slides and stained with hematoxylin and eosin (H&E) for histopathologic assessment. Stained sections were then scanned using an Aperio brightfield slide scanner (Leica Biosystems Inc., Richmond, IL, USA). Histopathology was evaluated by a board-certified veterinary pathologist.

### Quantitative assessment of histopathology and image slice registration

Correspondence between histopathology and in vivo imaging was assessed using the Dice similarity coefficient (DSC). The DSC describes the extent of spatial overlap between two binary image segmentations whereby 0 indicates no overlap and 1 indicates complete overlap [25]. A radiologist (MMH) selected the in vivo CT or MR imaging slice that best corresponded to the histopathology section using the position of the tissue within the mold and anatomic landmarks as guides. After a delay of > 1 month to effectively blind the reader, the radiologist contoured the perimeter of the liver and/or tumor in the previously identified CT or MR imaging slice, while blinded to the corresponding histopathology section. Digitized histopathology sections were independently contoured by an imaging scientist (ASM) and scaled to adjust for differences in pixel size relative to CT or MRI image slices. Binary masks were automatically generated based on the contours of histopathology and image slices using Matlab (Mathworks, Natick, MA). Corresponding masks were then overlaid and fine adjustments to the rotation of the histopathology mask were made if necessary. The largest linear dimension of in vivo and histopathology image masks was calculated in order to assess shrinkage of specimens associated with tissue explantation and processing. Finally, the DSC was calculated and reported as the mean DSC per specimen from 5–8 corresponding histopathology-imaging slice pairs (determined by the number of slots in the mold).

## Results

Specimen-specific 3D molds were generated and used to facilitate co-localization of histopathology sections with in vivo imaging. Liver specimens from five woodchucks were used to create five 3D molds using either contrast enhanced CT (N = 4) or MRI (N = 1) (Table 1). Two molds were created with cutting slots aligned with the axial imaging plane, and three molds had cutting slots aligned with the sagittal plane. Sectioning in the coronal plane was avoided since the resulting tissue sections would have been too large to fit on microscope slides. Two specimens were fixed in formalin prior to sectioning and three were sectioned fresh, immediately after euthanasia and resection. The length of time required for printing the 3D molds was between 9–23 hours and was largely dependent on the size of the mold.

**Table 1:** Study parameters and degree of spatial overlap of histopathology and *in vivo* imaging.

Woodchuck	Imaging modality	Specimen type	Preparation for sectioning	Days since imaging ^a^	Section plane	DSC ± SD [range]
1	CT	Tumor	Fixed	0	Axial	0.85±0.05 [0.78–0.89]
2	MRI	Tumor	Fixed	0	Axial	0.85±0.05 [0.77–0.91]
3	CT	Lobe	Unfixed	2	Sagittal	0.81±0.06 [0.74–0.91]
4	CT	Whole liver	Unfixed	2	Sagittal	0.82±0.02 [0.80–0.86]
5	CT	Whole liver	Unfixed	2	Sagittal	0.81±0.07 [0.73–0.89]

^a^ Refers to the time elapsed between imaging acquisition for mold design and specimen; DSC, Dice similarity coefficient.

**Fig 3:**
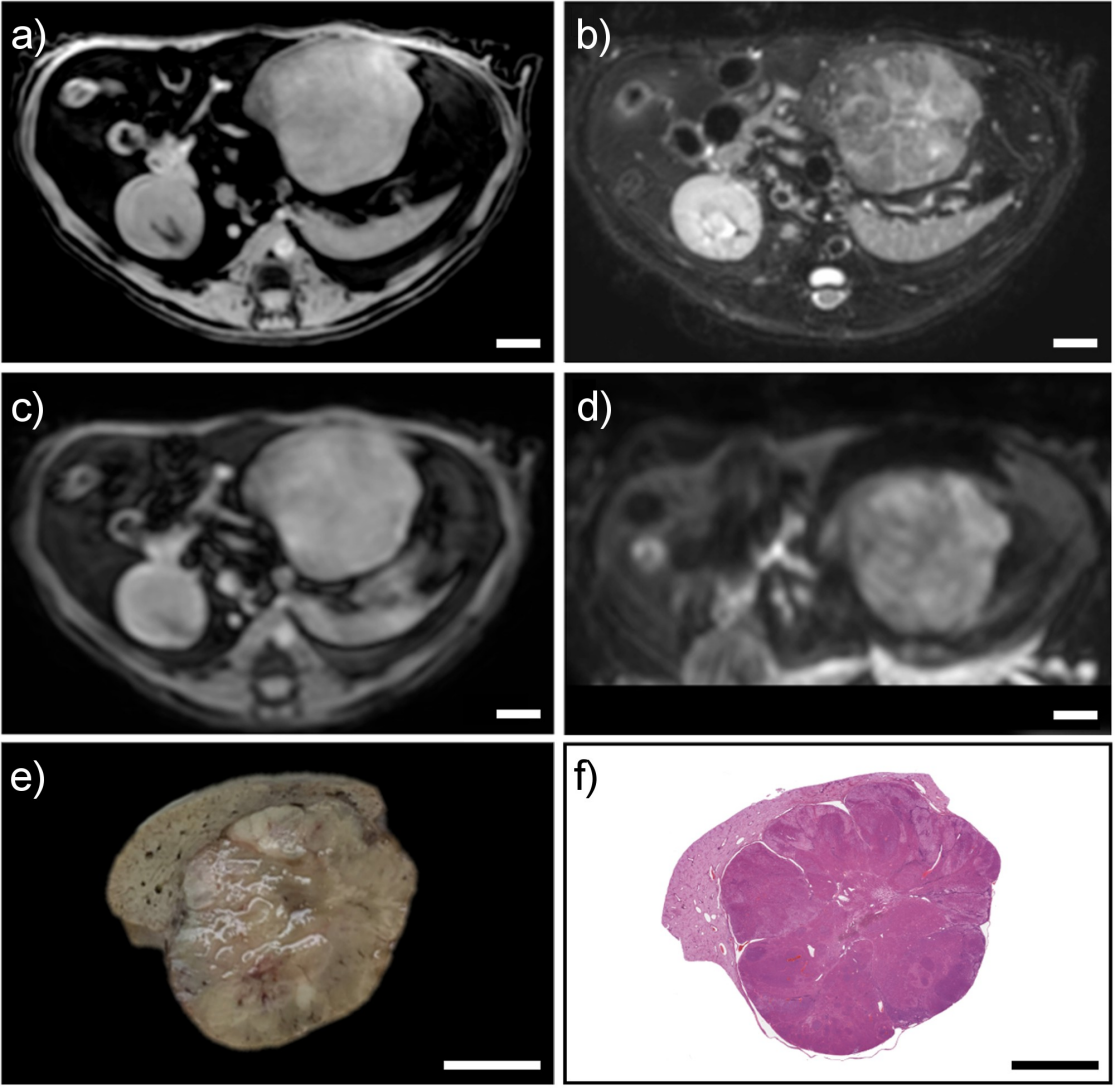
Corresponding *in vivo* MRI, gross pathology, and histopathology for a section from a formalin-fixed specimen. Multiple *in vivo* MRI sequences showing a large hepatic tumor, including: (A) T1 High Resolution Isotropic Volume Excitation (THRIVE), B) T2-weighted turbo spin echo, (C) dynamic contrast-enhanced, and (D) diffusion-weighted imaging. Formalin-fixed gross pathology section from the mold (E) with corresponding histopathology section (F) corresponding to slice location in MR images. Scale bars represent 1 cm.

**Fig 4:**
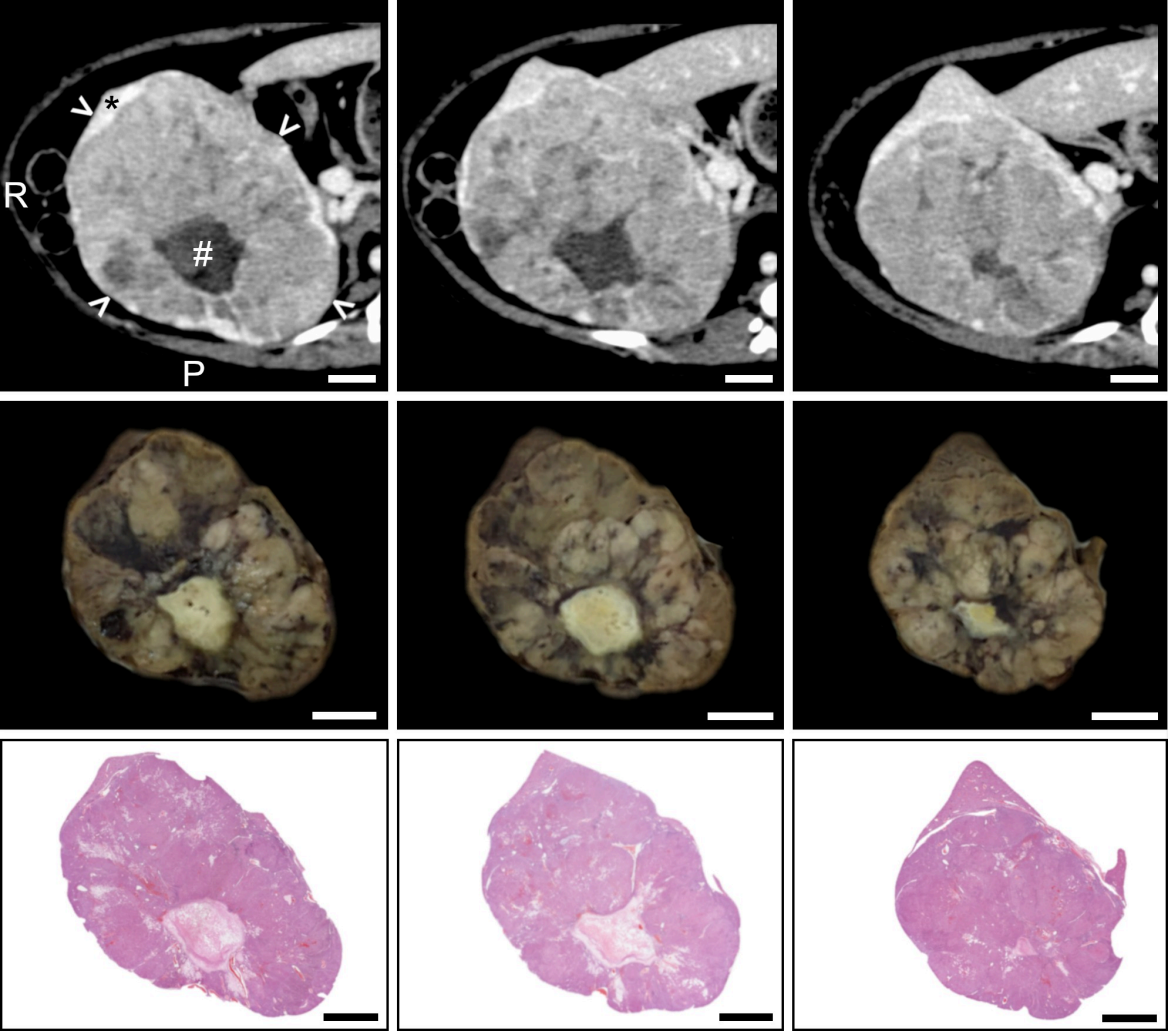
Sequential sections from a formalin-fixed tumor specimen with corresponding *in vivo* CT imaging and histopathology. Columns contain sequential axial gross pathology sections from the mold and corresponding CT and histopathology. (Top row) *In vivo* axial CT images acquired at the portal venous phase, showing a large tumor (arrowheads) containing central necrosis (#) with a small area of normal liver (*) visible at the anterior aspect. “R” and “P” represent right and posterior aspects, respectively. (Middle row) Gross pathologic specimens of the explanted tumor after sectioning using a 3D mold corresponding to the *in vivo* CT image slices. (Bottom row) Histopathology sections of the gross pathologic specimens. Correspondence of features can be seen between CT images of the hepatic tumor, gross pathologic specimens, and histopathologic specimens. Scale bars represent 1 cm.

**Fig 5:**
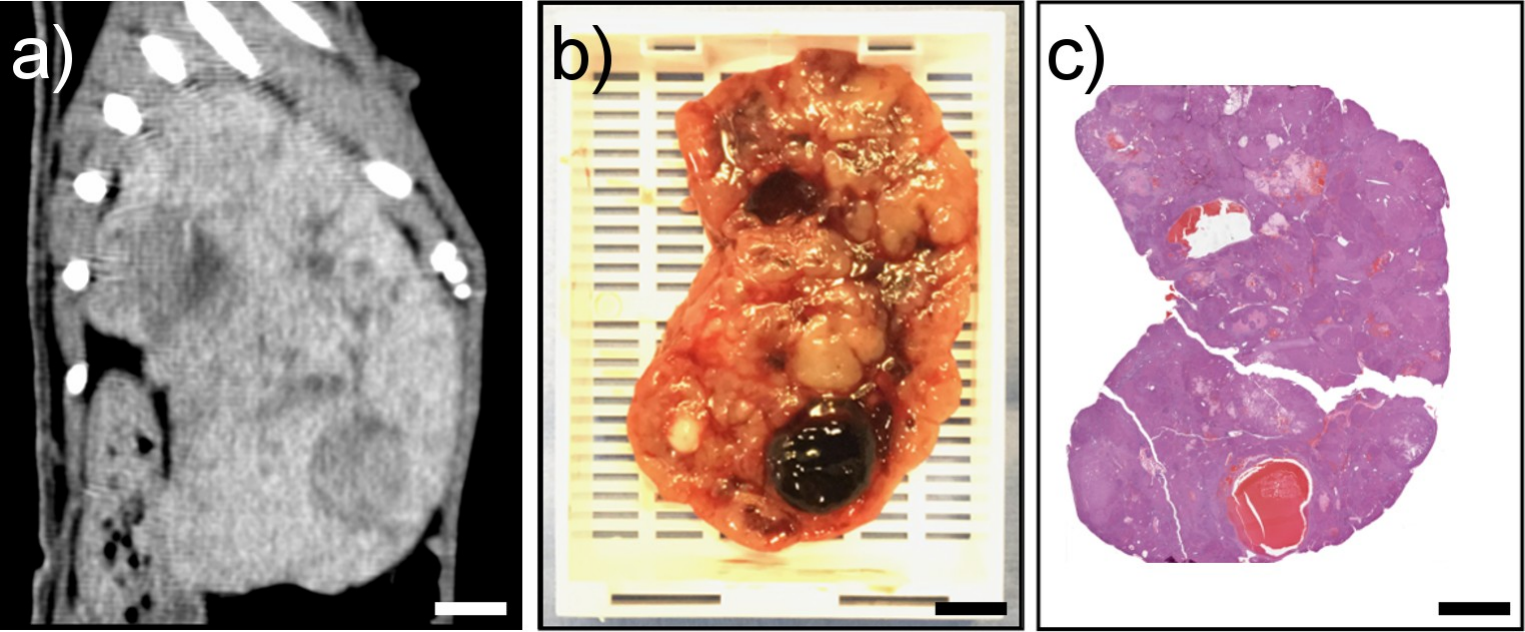
Corresponding *in vivo* CT imaging, gross pathology, and histopathology of a specimen cut without fixation. (A) CT slice in sagittal plane corresponding to gross- and histo-pathology tumor specimens in B and C. (B) Unfixed 5 mm thick gross pathology specimen from the mold. (C) Histopathology section stained with H&E. Scale bars represent 1 cm.

Accurate correspondence of imaging slices with gross pathology sections cut with (Figs 3 and 4) and without (Fig 5) prior fixation as well as histopathology reflect the excellent fit of the specimens within the mold cavity. Pathologic features can be seen in histopathology sections that correspond to features present in corresponding gross pathology sections and in vivo image slices where the specimen had been fixed prior to sectioning (Figs 3 and 4). A representative section from a specimen cut prior to fixation is shown demonstrating good correspondence between imaging, gross pathology and histopathology (Fig 5). Histopathology sections were an average of 15.3% smaller in largest linear dimension compared to corresponding regions on in vivo imaging. The mean DSC for all specimens was 0.83+/-0.05. Mold and specimen preparation parameters as well as the DSC for individual liver and tumor specimens are summarized in Table 1.

## Discussion

Accurate co-localization of in vivo imaging of the liver with histopathologic sections is challenging since the anatomic orientation and conformation of en bloc liver specimens is typically lost following resection. In our study, we assessed the use of liver-specific 3D cutting molds for co-localization of histopathology with in vivo MRI and CT using a woodchuck hepatic tumor model. We found that the molds provided accurate registration and orientation of histopathology sections with in vivo imaging as evidenced by a high degree of spatial overlap and correspondence of anatomic features.

The use of specimen-specific 3D molds for tissue sectioning serves two primary purposes: 1) to cut the specimen into tissue blocks of consistent thickness and spacing; and 2) to align the specimen with its original in situ orientation and 3D configuration relative to in vivo imaging such that sectioning allows accurate registration of imaging with histopathology. For highly deformable organs like the liver, the use of a mold that fully encases the specimen additionally serves to resist deformation during sectioning for improved fidelity to the imaging plane and subsequent registration of imaging and histopathology.

Depending on the application, formalin fixation may be desired for tissue preservation or unfixed specimens preferred if immediate tissue processing is required. Tissue fixation can cause changes in the specimen’s size during tissue processing and histologic slide preparation [26, 27]. We observed tissue shrinkage following formalin fixation resulting in a slightly looser fit inside the mold of fixed specimens compared to unfixed specimens. However, formalin fixation provided additional rigidity to the specimens which may have helped to mitigate deformation of pliable parenchyma during slicing. Shrinkage of slide-mounted histopathology sections was evident relative to corresponding tissues on in vivo imaging. This phenomenon may be attributed to the net effect of blood loss during resection, tissue fixation, and histologic tissue processing [28, 29]. Overall, slicing of formalin fixed or unfixed specimens with the molds resulted in good spatial overlap and correspondence of anatomic features between gross pathology, histopathology, and imaging.

The fidelity of image segmentation with respect to the contour of the resected specimen is an important determinant of a specimen’s fit inside the mold. However, resection boundaries cannot always be clearly demarcated on imaging a priori for mold design. Therefore, it was advantageous to perform the segmentation along a clear anatomic boundary, such as the perimeter of a pedunculated tumor, liver lobe, or entire liver, in such a way as to minimize the need for segmentation based on estimated and less predictable surgical margins. We performed lobar image segmentations and resections for tumors embedded in the liver parenchyma when lobe margins were conspicuous on imaging. Alternatively, whole liver image segmentation and explantation were performed. For pedunculated tumors, the tumor alone was segmented and resected.

Adequate time for mold production must be allotted between image acquisition and tissue resection for mold design and production if immediate sectioning of the specimen is desired. Potential morphologic changes, such as tumor growth or responses to therapy, should also be considered when determining the amount of time to allocate between imaging and specimen collection. In our study, image segmentation and mold design took approximately 3–4 hours, while printing took between 9 and 23 hours to complete and was largely dependent on the size of the mold. We found that acquiring imaging two days in advance of resection for immediate sectioning without fixation was sufficient time for mold production without compromising the fit of the specimens inside the mold cavity.

Hepatocellular carcinoma commonly manifests as multiple heterogeneous intrahepatic lesions with potentially variable sensitivity to treatment. Correlation of tissue and imaging features might enable development of AI prediction models that could enhance imaging-based molecular, genomic, or immunologic characterization of hepatic lesions leading to improved therapy selection or targeting. Such tissue-to-image mapping is particularly relevant for co-localization of histopathology with imaging findings in the setting of high specimen heterogeneity or multifocal disease. However, when the liver is resected it is no longer constrained to its in vivo configuration and undergoes significant deformation making correspondence with imaging challenging. By placing the explanted liver into a liver-specific 3D mold prior to sectioning, the in situ anatomic configuration is restored. Unlike conventional freehand tissue sectioning techniques, the use of 3D molds enables acquisition of serial gross pathology sections with uniform thickness and angulation that are registered with standard anatomic imaging planes.

Our study demonstrates that specimen-specific 3D molds can provide spatial co-localization of in vivo MRI or CT with gross- and histo-pathology specimens of whole-liver explants as well as resected liver lobes and tumors. We found that formalin fixation of tissue specimens was not necessary prior to sectioning in the molds, providing the option for immediate post-procedural tissue processing. We anticipate that the techniques described herein could be applied for sectioning human liver specimens following partial hepatectomy or liver transplantation based on pre-procedural CT or MRI in order to facilitate correspondence of imaging with histopathologic findings. Use of 3D molds within a clinical workflow would require adequate training or access to expertise in computer aided design, as well as greater time and expense relative to conventional tissue processing.

The techniques described in our study have several limitations. 3D molds must be created for each specimen, adding to costs and labor associated with tissue sectioning. Mold production requires access to a 3D printer and can cost in the range of 50 to 110 USD in materials depending on the size and design of the mold. However, the length of time and cost of production will depend on the model of printer and type of material used. Use of a single blade for tissue slicing may have contributed to some deformation or shifting of specimens inside the mold which could be minimized in the future by using a multi-blade knife. Spatial overlap between histopathology sections and image slices was diminished due to shrinking of the specimens resulting from blood loss upon resection, tissue fixation, and histopathologic staining. Comparisons were further complicated by the difference in slice thickness of in vivo images (1.5 mm for MRI and 0.8 mm for CT) relative to histopathology sections (5 μm in this study). For these reasons, we would not expect the image slices to exactly overlap with or match the appearance of gross pathology or histopathology sections. Nevertheless, the consistent angulation and spacing of tissue blocks within the molds with respect to in vivo imaging allowed for reasonably accurate and sequential pairing of histopathology sections with in vivo image slices.

## Conclusions

3D liver- and tumor-specific molds can facilitate accurate registration of in vivo imaging with histopathology, with or without tissue fixation prior to sectioning. This technique may be used for spatial correlation of imaging features or radiomic data with histopathology, immunohistochemistry or genomic analyses of the liver. Such tissue-to-image correlations may enable implementation of AI approaches for imaging-based histopathologic detection and classification tasks.

## Supporting information

S1 Checklist The ARRIVE guidelines checklist. (PDF)

S1 Table Dice similarity coefficients (DSC) demonstrating the spatial overlap of histopathology sections and corresponding image slices using liver-specific 3D molds. (XLSX)
